# Early Surgical Management of Large Scalp Infantile Hemangioma Using the TopClosure^®^ Tension-Relief System

**DOI:** 10.1097/MD.0000000000002128

**Published:** 2015-10-30

**Authors:** Zhanyong Zhu, Xilin Yang, Yueqiang Zhao, Huajun Fan, Mosheng Yu, Moris Topaz

**Affiliations:** From the Department of Plastic Surgery (ZZ, YZ, HF, MY); Department of Otorhinolaryngology (XY), Renmin Hospital of Wuhan University, Wuhan, Hubei, People's Republic of China; and Plastic Surgery Unit (MT), Hillel Yaffe Medical Center, Hadera, Israel.

## Abstract

Infantile hemangiomas (IHs) are the most common benign vascular neoplasms of infancy and childhood. The majority do not need medical intervention. However, large ulcerated scalp IHs may lead to fatal bleeding as well as severe cosmetic disfigurement that indicate early surgical excision, inflicting substantial surgical risks, with short- and long-term morbidity.

The TopClosure Tension-Relief System (TRS) is an innovative skin stretching and wound closure-secure system that facilitates primary closure of relatively large skin defects. This system has been shown as a substitute for skin grafts, flaps, or tissue expanders.

We describe a case of a giant IH of the scalp usually requiring a complex surgical approach, which was immediately primarily closed applying the TRS.

A 3-day-old female infant presented with a giant scalp hemangioma at birth that rapidly grew in the neonatal period with early signs of ulceration. The patient underwent surgical resection of the giant scalp hemangioma with immediate primary closure of the defect using the TRS. Surgical procedure and postoperative period were uneventful.

Early surgical resections of IHs at infancy carry substantial surgical risks and morbidity. This is the first reported case of early resection of a scalp hemangioma in the neonatal period, with successful immediate primary closure by application of stress-relaxation technique through the TRS. The application of the TopClosure TRS in this age group has significant advantages. It reduces the complexity and length of surgery, reducing blood loss, eliminating donor site morbidity, improving wound aesthetics, and minimizing the need for future reconstructive procedures.

## INTRODUCTION

Infantile hemangiomas (IHs) are the most common benign vascular tumors in infancy, proliferating rapidly during the first year of life, and then gradually involuting thereafter. IHs are most frequently located in the head and neck region (60%), followed by the trunk (25%) and the extremities (15%).^1^ There are various options regarding the treatment of IHs such as pulsed dye laser treatment, oral steroids, and propranolol treatment that are suitable for most cases.^2^ Although most IHs involute and never require surgical intervention, some may cause life-threatening bleeding, severe cosmetic deformity, and threaten tissue integrity, indicating surgical excision. Large scalp IHs are often associated with serious sequelae such as ulceration, bleeding, cardiac failure, as well as long-term outcomes such as alopecia, conspicuous scarring, and even malformation of the cranial vault.^3^ When significant functional impairment, ulceration, bleeding, or cosmetic deformity is encountered or anticipated, surgery can be performed, if feasible, within 4 months of age.^4^

Owing to the potential serious surgical risks such as massive bleeding and difficulty in closing the skin defect primarily following the excision of large IHs, surgical resection is often hindered. In the present report, we aim to describe a case of a newborn who was treated by total surgical removal of a large scalp IH with immediate primary closure using the newly introduced TopClosure Tension-Relief System (TRS) and technique, allowing the application of both stress relaxation and mechanical creep for skin stretching.^5–7^ The system is composed of 2 flexible polymer attachment plates (APs) that are attached to the skin by adhesive or by the customary skin staples or sutures, over a large area of adherence. This is the first reported case of the application of this newly developed system in a newborn.

## CASE REPORT

**FIGURE 1:**
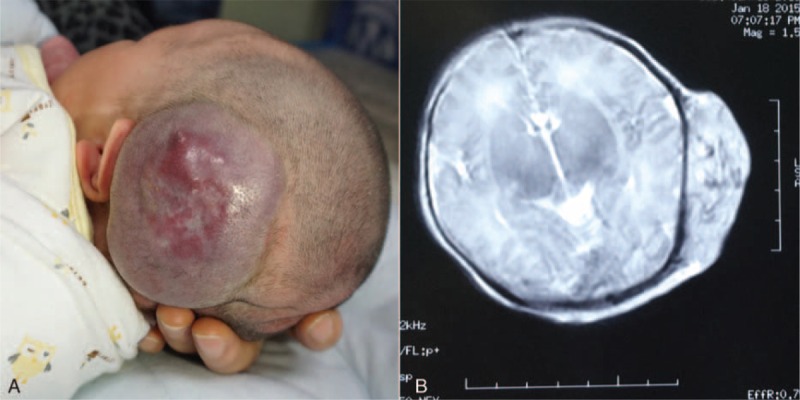
(A) After birth, large scalp hemangioma was in the left parietal region. (B) The extent of tumor was demonstrated on MRI image. MRI = magnetic resonance imaging.

An otherwise healthy female infant was born with a 4.8 × 4.0 cm mass on the left parietal region (Fig. 1A). Magnetic resonance imaging demonstrated a 5.0 × 4.0 cm homogeneous hemangioma that pressed the underlying skull inward with no other obvious abnormalities in the head (Fig. 1B). The lesion was slightly erythematous and warm, with no thrill felt on palpation. The fontanelle was soft and flat, and the remaining physical and neurological examinations were normal. The volume of the tumor increased rapidly in only 1 week, with early signs of necrosis at its apex. Other preoperative blood examinations were in the normal range. The electrocardiography and the echocardiographs were normal. Owing to the high risk for serious complications such as severe bleeding and permanent disfigurement, we decided to perform a total resection of the tumor.

### Operative Details

**FIGURE 2:**
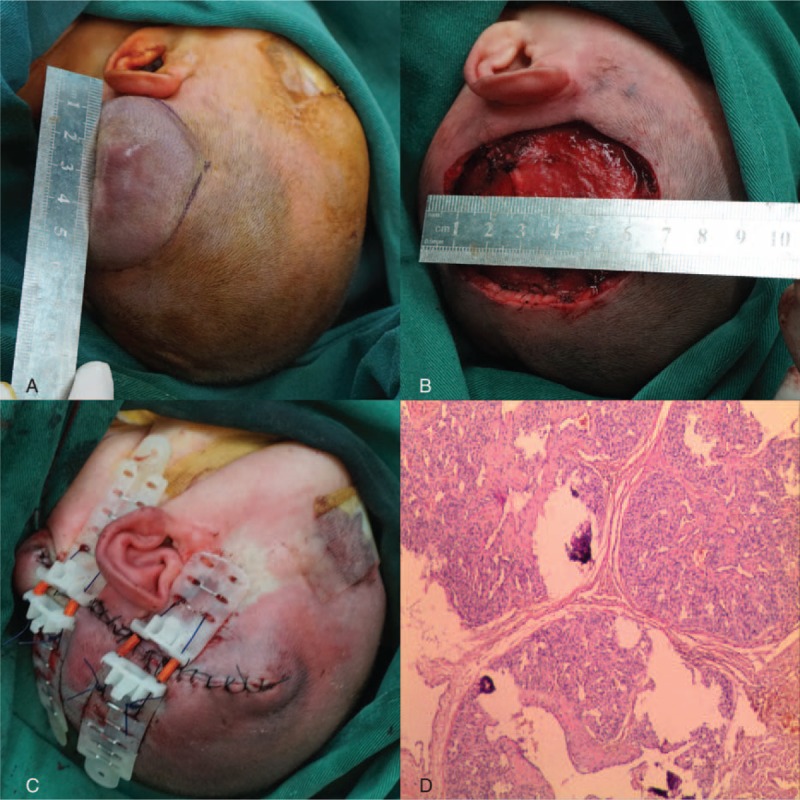
(A) Ten days later, the rapid growth of huge hemangioma in the left parietal region. (B) Tumor ablation resulted in a defect of about 6.5 × 5.2 cm. (C) Immediate primary closure was achieved using 2 TopClosure sets. (D) The pathological evaluation revealed a cavernous hemangioma.

A surgical resection was performed under general anesthesia when the patient was 10 days old. The surgical incision was marked along the borders of the tumor, measuring 6.2 × 5.1 cm (Fig. 2A). The numerous vascular pedicles of small arteries and veins associated with the lesion were meticulously divided and ligated. The hemangioma was completely removed resulting in a 6.5 × 5.2 cm soft tissue scalp defect, too large to be closed by a simple suturing technique (Fig. 2B). In order to avoid skin grafting or flaps, the TopClosure TRS (IVT Medical Ltd., Ra’anana, Israel) was applied for immediate primary wound closure. The surgical technique was previously described in detail by Topaz et al.^7^ Two pairs of APs were attached to the skin, 1.5 cm away from the wound edges, and secured by skin staples (Weck Visistat, Teleflex Medical, NC). Two tension sutures (Ethicon 0, MO-2 PDS∗ II, 40 mm 1/2C; Johnson & Johnson International, Inc. New Brunswick, New Jersey) were introduced through each AP. The needle was passed through the AP, deep over the periosteum and across the skin defect, and then out through the opposite AP on the contralateral side of the scalp defect. The suture was then passed back to the other side through the designated holes in the front part of the APs to the first plate. Multiple cycles of stress relaxation of tension application for 30 seconds and relaxation for 30 to 60 seconds were performed over a period of about 20 minutes for immediate primary approximation of the wound edges. Interrupted absorbable subcutaneous sutures (Ethicon 4-0, VICRYL∗Plus Antibacterial, 22 mm 1/2C; Johnson & Johnson International) were applied concurrent with the pull on the tension sutures to meticulously obliterate dead space. As no undermining of wound edges was performed, dead space was negligible and no drain was required. Wound edges were aligned by interrupted silk sutures (Ethicon MERSILK 5-0; Johnson & Johnson International) (Fig. 2C). The entire surgical procedure lasted for about 2 hours, achieving immediate primary wound closure with estimated blood loss being <20 cc and the patient was hemodynamically stable throughout the entire procedure.

### Follow-Up and Outcome

**FIGURE 3:**
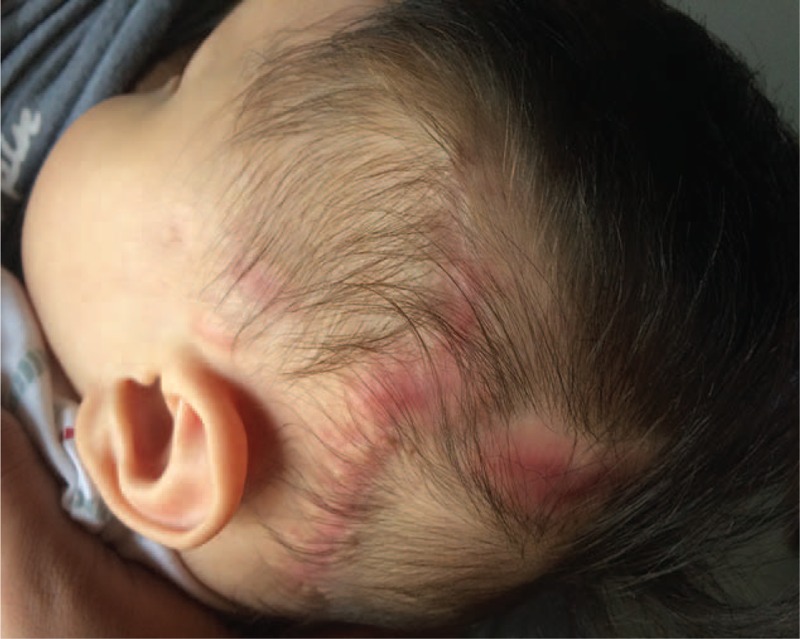
Six months of follow-up after surgery, the acceptable aesthetic result, mild partial hypertrophic scar, and linear alopecia of the scalp scars.

The pathological evaluation revealed a cavernous hemangioma (Fig. 2D). The TRS was applied for 14 days following the surgery to ensure wound closure. There was no ischemia or necrosis of the wound edges, in spite of the high-tension closure, and the infant tolerated the whole procedure well. At follow-up evaluation of the surgical scar after 6 months, the wound was completely closed, with an aesthetically acceptable, minimally depressed scar (Fig. 3).

An ethical committee approval for the above reported procedure was waived by the Medical Ethics Committee of the Renmin Hospital of Wuhan University Review Board, as a single case study was not considered to be “research.” Following a detailed review of the intended surgical procedure, the parent's informed consent was granted, together with their later written approval of the publication of this case report manuscript.

## DISCUSSION

Infantile hemangiomas are the most common tumors of infancy that affect up to 2% of full-term newborns.^8^ IHs often proliferate rapidly during the first few weeks of life, and approximately 80% of the superficial hemangioma growth is achieved by 3 months of age.^9^ Various therapeutic approaches have been described, such as laser treatment, systemic or intralesional steroids, systemic propranolol, and others. Oral propranolol has received more interest than other therapies in recent years. Since the initial report by Léauté-Labrèze et al,^10^ many studies have confirmed the beneficial effects of propranolol in treating IHs.^11,12^ Although surgical excision is not the first choice of treatment in most cases, it should be considered in cases of large IHs, when ulceration, bleeding, or hemodynamic instability occurs, and when life-threatening complications as well as substantial permanent contour deformity during the evolution of the IHs are anticipated. Even following regression, large IHs may also leave behind fibrofatty tissue along with alopecia or a poor cosmetic outcome that can cause psychological stress to patients as well as their parents.^4^ In our case, the newborn presented to our clinic with a large scalp hemangioma that was in a highly proliferating phase. The early necrosis on its apex indicated the risk for bleeding that could threaten the patient's life. All these indicated the need for early surgical intervention for this neonate. The main concern in performing surgery for IHs in the newborn is the consequences of blood loss, as even small amounts of bleeding could not be compensated at this age, resulting in life-threatening conditions. Careful ligation with suture of the major vessels and bipolar coagulation of the small vessels are essential for the control of bleeding as well as the prevention of the hematoma formation after surgery.^13^

After surgical resection, careful primary closure of the wound is of utmost importance. Direct approximation by suturing is not suitable for the 6.5 × 5.2 cm scalp defect resulting from surgery. Skin grafts or flaps that have been previously proposed for the reconstruction of these defects would have caused greater surgical trauma, risk of bleeding, infection, and a longer operative time that would be hard to endure for a 10-day-old newborn. The TopClosure TRS, first reported in 2012 by Topaz et al, is an innovative device for wound closure.^5,7^ It provides a flexible, secured attachment to the skin in a large area of attachment, without undermining, keeping intact blood supply to wound edges. This aids closure of small to huge wounds with significant loss of skin and soft tissue, through en bloc mobilization of skin and subcutaneous tissue, through relatively simple procedure, thus significantly downgrading surgical complexity.

There is a significant advantage in the use of TopClosure in the pediatric group of patients for revision of scars and for the excision of big masses, by reducing the need for serial excisions as a substitute for tissue expanders and for the alleviation of pain when used noninvasively. Moreover, the application of this device did help in reducing the blood loss, for there was no need to dissect and undermine the subcutaneous tissues around the defect lesion to ease the closure procedure.

The Top Closure system remained attached to the skin for 14 days after surgery, in order to help secure the wound edges and improve scar aesthetics. The patient returned to the hospital for a follow-up 6 months later, with an excellent cosmetic outcome with minimal alopecia. This case is the youngest patient who has been treated with the TopClosure system. The surgical success of this procedure indicates that closing large defects with the TRS is an excellent option, especially for infants.

## CONCLUSIONS

Although there is no consensus on the optimal age for surgical resection of large scalp IHs, some researchers have recommended early excision (<4 months of age).^3^ With the developments in surgical techniques and anesthesia management, age should rarely be an obstacle for treating patients with larger IHs requiring surgical intervention. Application of the TopClosure system can be used to modify and improve the current practice of wound closure.
